# Blockade of the Notch Signaling Pathway Promotes M2 Macrophage Polarization to Suppress Cardiac Fibrosis Remodeling in Mice With Myocardial Infarction

**DOI:** 10.3389/fcvm.2021.639476

**Published:** 2022-01-17

**Authors:** Zhi Li, Miao Nie, Liming Yu, Dengshun Tao, Qiang Wang, Yuanchen He, Yu Liu, Yuji Zhang, Hongguang Han, Huishan Wang

**Affiliations:** ^1^Department of Cardiothoracic Surgery, General Hospital of Northern Theater Command, Shenyang, China; ^2^College of Animal Science and Veterinary Medicine, Shenyang Agricultural University, Shenyang, China

**Keywords:** Notch signaling pathway, myocardial infarction, fibrotic remodeling, CYLD expression, macrophage polarization, inflammation

## Abstract

Myocardial infarction (MI) is regarded as a serious ischemic heart disease on a global level. The current study set out to explore the mechanism of the Notch signaling pathway in the regulation of fibrosis remodeling after the occurrence of MI. First, experimental mice were infected with recombination signal binding protein J (RBP-J) shRNA and empty adenovirus vector, followed by the establishment of MI mouse models and detection of cardiac function. After 4 weeks of MI, mice in the sh-RBP-J group were found to exhibit significantly improved cardiac function relative to the sh-NC group. Moreover, knockdown of RBP-J brought about decreased infarct area, promoted cardiac macrophages M2 polarization, reduced cardiac fibrosis, and further decreased transcription and protein expressions of inflammatory factors and fibrosis-related factors. Furthermore, downregulation of cylindromatosis (CYLD) using si-CYLD reversed the results that knockdown of RBP-J inhibited fibrogenesis and the release of inflammatory factors. Altogether, our findings indicated that the blockade of Notch signaling promotes M2 polarization of cardiac macrophages and improves cardiac function by inhibiting the imbalance of fibrotic remodeling after MI.

## Introduction

Myocardial infarction (MI), precipitated by the blockade of a coronary artery, ranks as one of the leading causes of mortality and disability across the world (1). Physiologically, initial ischemia is followed by an acute inflammatory response, which results in neutrophil infiltration in the necrotic area and subsequent macrophage infiltration (2). In addition, cardiac fibrosis is established as a common manifestation of cardiac remodeling after MI, whereas the phenomenon of excessive remodeling of myocardial fibers can precipitate a downhill cascade, leading to chronic heart failure and death (3).

Unsurprisingly, macrophages are often implicated in the inflammation that ensues after MI (4). More precisely, the M1 form of macrophages is known to promote the progression of inflammatory responses, whereas M2 macrophages exert a certain anti-inflammatory effect and a promoting effect on the proliferation of endothelial cells and fibroblasts, thus collectively regulating the immune microenvironment at the site of infarction by influencing their functional characteristics (5). In addition, there is evidence to suggest the important regulatory role of macrophage polarization in cardiac remodeling after MI in humans (6). Nevertheless, the underlying regulatory mechanism and potential effects of macrophage polarization concerning MI remain unknown.

As for another key focus of our study, recombination signal binding protein (RBP)-J is established as an important transcription factor downstream of the Notch signaling pathway and further possesses the ability to mediate the main transactivation signals of Notch receptors (7). This is particularly important as the Notch pathway mediated through RBP-J was previously shown to confer regulatory roles on cell growth, differentiation, apoptosis, and remodeling after tissue injury, while it is also implicated in critical processes such as macrophage activation and plasticity (8, 9). In addition, existing evidence highlights the correlation between M2 polarization of macrophages and Notch signaling blockade (7). Moreover, evidence has suggested that overexpression of Notch signaling might exert fibrogenic influences on different diseases, such as kidney fibrosis, scleroderma, and cardiac fibrosis (9, 10). Meanwhile, regarding MI, the Notch pathway is also known to play various roles in MI. For instance, one study revealed that the Notch pathway is associated with cardiac repair and regeneration after MI (11), while the activation of the Notch pathway was shown to inhibit ventricular remodeling in MI rats (12). Similarly, an early study illustrated that Notch was activated during post-MI remodeling (13), which reiterates the important role of the Notch pathway in post-MI cardiac remodeling. However, whether the Notch signal mediates the polarization of macrophages to influence the fibrosis remodeling process of MI requires further exploration.

Coronary artery occlusion after MI is further associated with tissue hypoxia, myocardial cell necrosis, and the release of a large number of inflammatory chemokines, all of which lead to substantial inflammatory cell infiltration at the site of infarction (14, 15). Meanwhile, the study performed by Elsser *et al*. highlighted the involvement of the cylindromatosis (CYLD) gene in the regulation of the NF-κB pathway, while also exhibiting a close association with cellular inflammation and fibrosis (16). Furthermore, blockade of the Notch pathway was previously indicated to inhibit NF-κB activation by promoting the expression of CYLD in ischemia-reperfusion (17). However, it remains unclear whether the Notch signaling can mediate macrophage polarization to regulate myocardial fibrosis after MI and whether the regulation is related to the CYLD gene. Based on the aforementioned evidence, the current study set out to explore the mechanism of the Notch signaling pathway in the regulation of macrophage polarization in fibrosis remodeling after MI, to uncover a novel treatment strategy against cardiac remodeling in MI.

## Materials and Methods

### Experimental Animals

First, adult male C57BL/6 mice (6–8 weeks old, weighing 20–25 g) were procured from Shanghai SLAC Laboratory Animal Co., Ltd. (Shanghai, China). The obtained mice were housed in a clean environment at 20°C for 12 h, with alternating light and dark and humidity conditions of 50–60%. All animal procedures were performed following the Guidelines of the Animal Ethics Committee of General Hospital of Northern Theater Command. Extensive efforts were undertaken to minimize both the number and suffering of the experimental animals.

### Establishment of MI Models in Mice

The MI mouse models were established by ligating the anterior descending coronary artery in mice hearts. Briefly, the mice were anesthetized with 1.0–1.5% isoflurane, intubated, and then connected to a ventilator for small animals. Subsequently, the thoracic cavity of mice was opened from the 4th intercostal space and the pericardial wall was bluntly separated. Next, the anterior descending coronary artery was ligated using a 7-0 silk thread, and the whitening of myocardial tissue in the ligated area was considered indicative of successful modeling. The thoracic cavity was then closed with a 5-0 silk thread. Afterward, the mice were allocated into the MI group (model group) and the sham group. Mice in the sham group were not subjected to ligation of the anterior descending coronary artery, and the other treatments were the same as the MI group. Three days before the establishment of the MI models, the mice were injected with adeno-associated virus (AAV2)-packaged RBP-J shRNA and adeno-associated virus-packaged sh-NC (5 × 10^9^/100 μl) into the apex of the heart, and regarded as the sh-RBP-J group and the sh-NC group, respectively. Four weeks later, after determining the cardiac function, all animals were intraperitoneally euthanized with pentobarbital sodium (≥ 100 mg/kg), followed by heart removal. A total of 8 mice from each group were used for the section preparation, with the hearts embedded in paraffin and sliced into 4-μm sections for subsequent experimentation. Additionally, 6 mice were employed for protein determination experiments, 6 mice for flow experiments, and 3 mice for 2,3,5-triphenyl tetrazolium chloride (TTC) detection.

### Measurement of Cardiac Function

All mice were subjected to echocardiography after 4 weeks of MI to evaluate cardiac function. Briefly, pentobarbital sodium (40 mg/kg) was intraperitoneally administered to induce anesthesia, and the mice were fixed on the examination table in a supine position. Subsequently, the hair was removed from the chest and an electrocardiogram was recorded simultaneously. M-mode ultrasound was further carried out to measure the left ventricular end-systolic diameter (LVES) and left ventricular end-diastolic diameter (LVED) in the parasternal long-axis view. Left ventricular ejection fraction (LVEF) and left ventricular fractional shortening (LVFS) were accordingly calculated and analyzed using the Vevo 2100 micro-ultrasound machine (VisualSonics, YTO, CAN).

### Measurement of Myocardial Infarct Size

Myocardial infarct size in mice hearts was measured using TTC staining. Four weeks after ligation, heart tissues were harvested after the mice were anesthetized and immediately frozen at 20°C for 5 min. The obtained hearts were sliced into five sections (3 mm) from the apex to the base. Subsequently, the obtained sections were placed in prewarmed 2% TTC solution (Sigma, St. Louis, United States) for 15 min at 37°C in conditions void of light, then fixed with 4% paraformaldehyde for 30 min, and photographed. Afterward, the stained areas were measured using the Image J analysis software (National Institutes of Health, Bethesda, Maryland, United States). The myocardial infarct size was calculated using the following formula: myocardial infarct size (%) = ∑ (myocardial infarct size per section/myocardial area per section × myocardial weight per section)/left ventricular weight × 100%.

### Terminal-Deoxynucleotidyl Transferase/(TdT)-Mediated Nick-End Labeling (TUNEL) Assay

Cell apoptosis in heart tissues was detected with the help of TUNEL assay detection kits (Roche, Basel, Switzerland) according to the manufacturer's instructions. Briefly, the heart sections were permeabilized with proteinase K solution for 15 min and then immersed in PBS. Subsequently, the enzymatic reaction was terminated with the addition of a termination reagent. Next, the sections were rinsed with PBS and incubated with streptavidin–horseradish peroxidase solution under conditions void of light for 30 min. After washing, the slides were immersed in 3, 3-diaminobenzidine and hematoxylin, followed by counting the number of TUNEL-positive cells and total cells.

### Histological Observation

Heart sections were deparaffinized and hydrated prior to hematoxylin–eosin (HE) staining. Hematoxylin staining was performed for 5 min and eosin staining for 5 s, followed by dehydration, transparency, neutral gum mounting, and finally observation and filming under a microscope (Olympus).

Masson staining was performed as follows: The prepared sections were sequentially treated with Masson staining solution (Sigma, St. Louis, United States), 1% phosphotungstic acid solution, aniline blue solution, and 1% glacial acetic acid solution. Subsequently, the sections were subjected to image collection and analyses after dehydration and clearing. Next, the prepared paraffined sections of mouse heart samples were collected, deparaffinized, repaired in an antigen retrieval solution water bath, dripped with normal goat serum-blocking solution (C-0005, Shanghai Haoran Biotechnology Co., Ltd., Shanghai, China), and then placed at room temperature for 20 min; afterward, primary rabbit-anti-mouse F4/80 (ab6640, dilution ratio of 10 μg/ml, Abcam, Cambridge, MA, United States), myeloperoxidase (MPO) (ab208670, dilution ratio of 1:1000, Abcam), and p65 (ab16502, dilution ratio of 1 μg/ml, Abcam) antibodies were added overnight at 4°C; the following day, goat anti-rabbit IgG (dilution ratio of 1:1000, ab6785, Abcam) secondary antibody was added and allowed to stand at 37°C for 20 min. Later, color was developed with DAB (ST033, Guangzhou Whiga Technology Co., Ltd., Guangzhou, China), counterstained with hematoxylin (PT001, Shanghai Bogoo Biotechnology Co., Ltd., Shanghai, China) for 1 min, and washed with water; the sections were then returned to blue with 1% ammonia and washed with water, then mounted with neutral resin, and observed and photographed under a microscope.

### Hydroxyproline (HYP) Content Determination

Approximately 0.5 g of heart samples were weighed and placed into a glass tube, minced for digestion, and then subjected to the treatment according to the instructions of the Hydroxyproline Assay kits (RampD SYSTEMS, Inc. MN, United States). The absorbance at a wavelength of 560 nm was detected using a visible spectrophotometer (Bio-Rad 680, Bio-Rad, Hercules, CA, United States).

### Macrophage Phenotyping

Nuclear translocation of p65 (ab16502, dilution ratio of 1 μg/ml, Abcam) was detected in cultured macrophages, and the samples were incubated at 4°C overnight. The following day, goat anti-rabbit antibody IgG (ab205718, dilution ratio of 1:2000, Abcam) containing tetramethylrhodamine isothiocyanate (TRITC) was added to the samples and stored at room temperature for 1 h. DAPI was then carried out to stain the nuclei, and the cells were subsequently observed under a Biorevo BZ9000 fluorescence microscope (Keyence, Osaka, Japan). In accordance with previous literature (18), a single-cell suspension or primary cultured macrophage suspension was prepared from the minced heart tissue block, which was diluted to 1 × 10^9^ cells/ml and placed in EP tubes. Subsequently, paraformaldehyde (2 ml 40 g/L) was added to the EP tubes, gently mixed, and placed in a 37°C water bath for 10 min. Next, the supernatant was discarded after centrifugation at 260 g for 5 min, and 1 ml of 90% methanol (precooled at 4°C in advance) was added, gently mixed, and placed in an icebox for 30 min. Afterward, the cells were collected after centrifugation at 260 g for 5 min and the supernatant was discarded. Thereafter, 300 μl of primary antibodies CD11b (ab8878, Abcam), iNOS (ab115819, Abcam), CD206 (ab125028, Abcam), F4/80 (ab6640, Abcam), and Gr1 (ab238132, Abcam) were all added to the cells, gently mixed, and then incubated for 1 h at room temperature in conditions void of light. The cells were collected after centrifugation at 260 g for 5 min and the supernatant was discarded, and then 300 μl of Alexa Fluor ® 488-labeled secondary antibody IgG (ab205718, dilution ratio of 1:2000, Abcam) was added and incubated for 30 min at room temperature in dark conditions, and finally detected with a flow cytometer (MoFloAstrios EQ, Beckman Coulter, Inc, CA, United States).

### Detection of Fibrosis-Related Factors and Inflammatory Factors

The ELISA was adopted to detect the contents of fibrosis-related factors TGF-β1, PDGF-B, COL1, and COL3 in mice heart tissues and the levels of TNF-α, IL-1β, and IL-6 in the serum according to the manufacturer's instructions (RampD SYSTEMS, Inc. MN, United States).

### Isolation and Identification of Bone Marrow-Derived Macrophages

Mice were soaked in 75% alcohol for 5 min after the induction of euthanasia. Subsequently, both ends of the tibia and fibula were cut off, and bone marrow cells were flushed out using a syringe needle and collected in a flat dish. A single-cell suspension was then prepared and seeded in a culture dish. Non-adherent cells were removed after 7 days, and adherent cells were regarded as bone marrow-derived undifferentiated macrophages. Afterward, macrophages were identified using flow cytometry.

### Isolation and Identification of Cardiac Fibroblasts

Mice hearts were removed after opening the chest with sterilized surgical scissors and placed in pre-cooled PBS. Subsequently, the extracted hearts were transferred to a super clean bench to cut off the atria and excess connective tissue, and the open-heart cavity was cut to remove blood clots and repeatedly rinsed with precooled PBS 3 ~ 4 times to remove the excess blood. Next, the ventricles were sliced into tissue pieces of about 1 cm^3^ in size and rinsed with precooled PBS until the liquid was clear, and the supernatant was then sucked up. A digestive solution (1 g/L trypsin, 0.8 g/L type IV collagenase) was prepared with Ca^2+^-free PBS and filtered for sterilization, and 6 ml of the digestive solution was added to the tissue block, followed by shaking at 100 g for 6 min in a 37°C water bath. Next, the supernatant was transferred to a 50-ml centrifuge tube, with equal volumes of DMEM added to terminate the digestion. The above steps were repeated three times. Afterward, the obtained cell suspension was filtered and centrifuged (4°C, 300g, 10 min), followed by the removal of the supernatant. The cell pellets were resuspended with 30 ml of DMEM containing 100 ml/L FBS, equally divided into culture dishes, and then cultured in an incubator at 37°C for 90 min. After differential adherence, the dish was shaken using the cross-method, and the cells were washed into adherent cells and discarded, then added with DMEM, and placed in an incubator for culture. The solution was changed every 3 days. Immunohistochemistry was subsequently performed to identify isolated fibroblasts. The cultured cells were isolated from the hearts, fixed with 4% paraformaldehyde for 15 min, and rinsed three times with PBS. After the cells were treated as per the instructions, primary antibodies vimentin (ab92547, dilution ratio of 1:300, Abcam), desmin (ab15200, dilution ratio of 1:200, Abcam), and vWF (von Willebrand factor) (AB7356, Sigma-Aldrich) were added and incubated at 4°C and overnight. The following day, after rewarming for 30 min, the secondary antibody (ab205718, Abcam) was added to the cells, followed by incubation at 37°C for 15 min, DAB development for 2 min, and observation under a light microscope.

### Co-Culture of Cells

Macrophages were co-cultured with cardiac fibroblasts using a Transwell chamber. Briefly, prior to the co-culture, the cardiac fibroblast culture medium was added with 1 μg/ml LPS and 20 ng/ml LIFN-γ (LPS + IFN-γ) and then cultured for 24 h. Subsequently, the fibroblast suspension (500 μl, 1 × 10^6^ cells/mL) was added to the upper chamber of the Transwell coated with Matrigel, while the macrophages (500 μl, 1 × 10^6^ cells/ml) were added to the lower chamber, containing RPMI-1640 cell culture medium with 10% fetal bovine serum, 1,000 U/ml penicillin, 100 mg/ml streptomycin, and 1 μg/ml LPS as the culture medium. Three replicate wells were prepared for each group and incubated at 37°C with 5% CO_2_ in the air for 48 h.

### Cell Transfection

Bone marrow-derived macrophage cells at the logarithmic phase of growth obtained from MI mice in the sh-RBP-J group and the sh-NC group were seeded in 6-well plates at a concentration of 5 × 10^4^ cells/ml and then placed in a culture chamber for routine culture. Upon reaching 70% cell confluence, the siRNA of CYLD was transfected into each cell group according to the instructions of the Lipofectamine^TM^ 2000 transfection reagent and regarded as the sh-RBP-J-siRNA group, sh-RBP-J-Scramble group, sh-NC-siRNA group, and sh-NC-Scramble group. After transfection for 6 h, the cells were cultured with a fresh culture medium and collected after 48 h. The expression patterns of CYLD protein in the cells were detected using a Western blot assay.

### Western Blot Assay

Total protein content was extracted from the cells using a RIPA lysis buffer containing PMSF (Beyotime, Beijing, China), and the protein concentration was determined using bicinchoninic acid (BCA) protein quantification kits (Wuhan Boster Biological Technology, Ltd., Wuhan, China). Subsequently, the obtained proteins were separated with 10% SDS–PAGE, then electrotransferred to poly(vinylidene fluoride) (PVDF) membranes, and blocked with 5% bovine serum albumin (BSA) for 2 h at room temperature to block the non-specific binding. Afterward, diluted primary antibodies rabbit anti-mouse RBP-J (ab180588, dilution ratio of 1:1000, Abcam), CYLD (ab137524, dilution ratio of 1:1000, Abcam), p-p65 (ab194726, dilution ratio of 1:500, Abcam), p-IκBα (ab133462, dilution ratio of 1:1000, Abcam), and β-actin (ab8227, dilution ratio of 1:2000, Abcam) were added, respectively, and incubated overnight at 4°C. The following day, the membrane was washed and incubated with the goat anti-rabbit secondary antibody IgG (ab205718, dilution ratio of 1:2000, Abcam) for 1 h at room temperature and then developed with the ECL solution (EMD Millipore, United States). The grayscale of bands in the Western blot images was quantified using the Image Pro Plus 6.0 software (Media Cybernetics, United States), with β-actin serving as an internal reference.

### Quantitative Real-Time PCR (qRT-PCR)

Total RNA content was extracted from the cells using the RNAiso Plus (TAKARA, Otsu, Shiga, Japan) and Trizol LS Reagent (TAKARA, Otsu, Shiga, Japan), respectively. Formaldehyde-denaturing electrophoresis detection was adopted to verify the reliability of the process. Reverse transcription experiments were subsequently performed with the help of PrimeScript^TM^ RT kits (TAKARA, Otsu, Shiga, Japan), in strict accordance with the manufacturer's instructions. The relative expression levels of the genes were quantified using the standard qRT-PCR with SYBR Premix Ex Taq (TAKARA, Otsu, Shiga, Japan), with β-actin serving as the reference gene, and primers for each group are shown in Table 1.

**Table 1:** Primers sequence.

**Gene**	**Forward**	**Reverse**
TGF-β1	GACCGCAACAACGCCATCTA	GGCGTATCAGTGGGGGTCAG
PDGF-B	TACCTGCGTCTGGTCAGC	GCTCGGGTCATGTTCAAG
COL1	GGTTGCAGCCTTGGTTAG	TGAGCCAGCAGATTGAGAA
COL3	GGTTTGGAGAATCTATGAATGGTGG	GCTGGAAAGAAGTCTGAGGAAGG
β-actin	CATGTACGTTGCTATCCAGGC	CTCCTTAATGTCACGCACGAT

### Statistical Analysis

All measurement data were analyzed using the SPSS 21.0 statistical software (IBM Corp. Armonk, NY, United States). Tests for normality and homogeneity of variance were performed to verify that the data were in a normal distribution with equal variance. Measurement data were expressed as mean ± SD. *T*-test was adopted for comparisons between two groups, and one-way ANOVA was used for comparisons between multiple groups, and Tukey's multiple test comparisons were performed for the *post-hoc* test. A value of *p* < 0.05 was regarded statistically significant.

## Results

### Blockade of the Notch Signaling Pathway Improved MI in Mice

**Figure 1:**
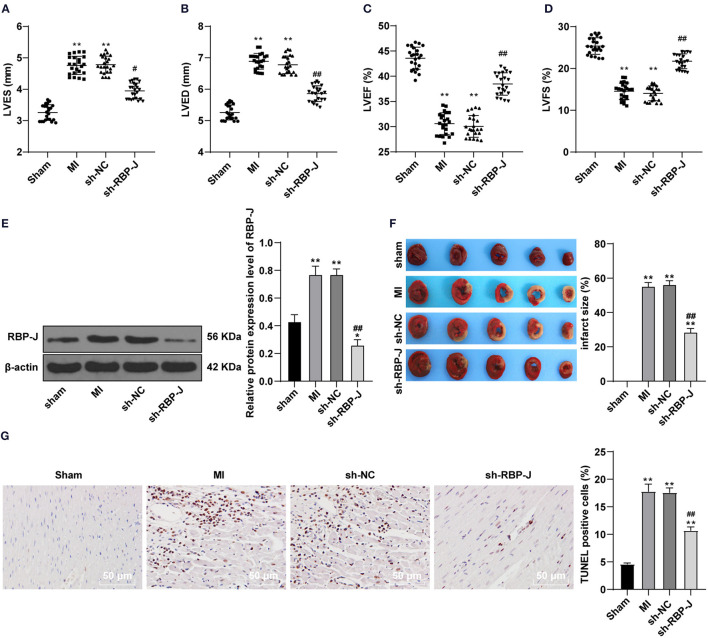
Blocking Notch signaling pathway improved myocardial injury in mice. **(A–D)**: Left ventricular end-systolic diameter (LVES), left ventricular end-diastolic diameter (LVED), left ventricular ejection fraction (LVEF), and left ventricular fractional shortening (LVFS) were analyzed by micro-ultrasound machine, *N* = 23/per group; **(E)**: Protein expression of RBP-J was detected, *N* = 6; **(F)**: Infarction area was detected by TTC method. **(G)**: TUNEL assay of the tissue apoptosis in the mouse heart. *N* = 3. Three independently repeated tests were performed, and the data were expressed as mean ±SD. One-way ANOVA was used for the analysis of variance, and Tukey's multiple comparisons test was used for the *post-hoc* test; ** *P* < 0.01 (compared with the sham group), ## *P* < 0.01, and # *P* < 0.05 (compared with the sh-NC group).

To verify the successful establishment of MI mouse models, cardiac function-related factors were first assessed. It was found that LVED and LVES were both significantly increased, while LVEF and LVFS were both markedly decreased in the MI group (all *P* < 0.05, Figures 1A–D), which indicated that the systolic and diastolic functions of the heart were significantly impaired due to coronary artery ligation, compounding to obvious cardiac dysfunction. Existing evidence further suggests that the Notch signaling pathway plays a significant role in the regulation of cell growth, differentiation, apoptosis, and remodeling after cardiac injury (19). Meanwhile, RBP-J is regarded as a key transcription factor downstream of the Notch signaling pathway. Accordingly, to investigate the involvement of Notch signaling in MI, we infected the MI mouse models with AAV-packaged RBP-J shRNA and AAV-packaged sh-NC and then detected the protein expression patterns of RBP-J in each group using a WB assay. Subsequent results demonstrated that there were no significant differences in RBP-J protein expression levels among the MI group and sh-NC group, while the RBP-J expression levels were significantly lowered in the sh-RBP-J group (*P* < 0.01, Figure 1E), suggestive of successful adenovirus infection. In addition, the results of the cardiac function test showed no significant differences between the sh-NC group and the MI group, while the sh-RBP-J group presented with improved cardiac function indexes (all *P* < 0.05, Figures 1A–D). Moreover, the myocardial infarct size was detected using TTC staining, the results of which illustrated that the myocardial infarct size was significantly lower in the sh-RBP-J group than that in the MI group and sh-NC group (*P* < 0.01, Figure 1F). Additionally, TUNEL staining illustrated that apoptotic cells were significantly decreased in the sh-RBP-J group compared with those in the MI group and sh-NC group (*P* < 0.01, Figure 1G). Altogether, these findings suggested that blocking the Notch signaling pathway improved myocardial injury in mice.

### Blockade of the Notch Signaling Pathway Promoted the M2 Polarization of Cardiac Macrophages and Reduced Fibrosis

**Figure 2:**
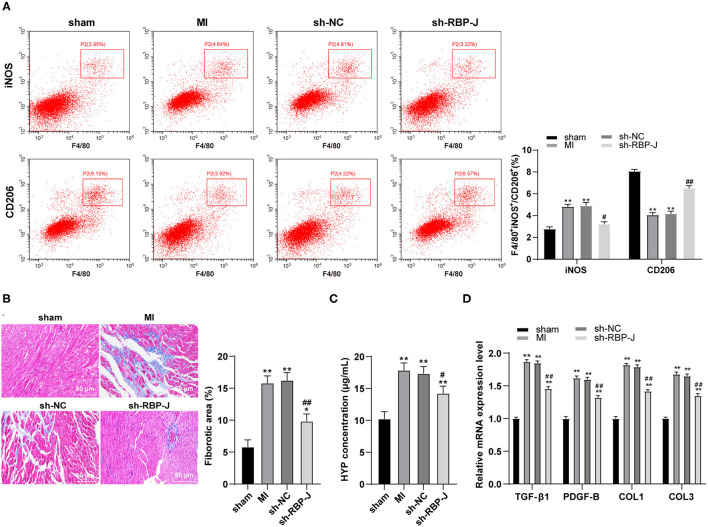
Blocking the Notch signaling pathway promoted the M2 polarization of cardiac macrophages and reduced the degree of fibrosis. **(A)**: Identification of macrophage types in the myocardial infarction area of mice by immunofluorescence and flow cytometry, *N* = 6; **(B)**: Observation of the degree of cardiac fibrosis in mice by MASSON staining, *N* = 8; **(C)**: Detection of HYP content in the heart by ELISA kit, *N* = 6; **(D)**: Detection of expression of TGF-β1, PDGF-B, COL1, and COL3 in the heart by qRT-PCR, *N* = 6; three independently repeated tests were performed, and the data were expressed as mean ± SD. One-way ANOVA was used for analysis of variance, and Tukey's multiple comparisons test was used for *post-hoc* test; ** *P* < 0.01, * *P* < 0.05 (compared with sham group), and ## *P* < 0.01, # *P* < 0.05 (compared with sh-NC group).

Previous studies have documented the participation of macrophages in the vast process of MI remodeling, while M1- and M2-type macrophages have also been found in the MI area (2, 20). Therefore, we hypothesized that Notch signaling regulated macrophage polarization and influenced fibrotic remodeling after MI. Subsequently, flow cytometry was carried out to identify the macrophage types in the MI area of mouse hearts, which revealed that the expression levels of CD206 (M2 macrophage marker) were upregulated, while those of iNOS (M1 macrophage marker) were downregulated in the sh-RBP-J group (all *P* < 0.05, Figure 2A), suggesting that blocking the Notch signaling pathway promoted M2 macrophage polarization. Furthermore, Masson staining was adopted to observe the degree of cardiac fibrosis in mice, the results of which illustrated that the collagen content and the degree of fibrosis were both significantly decreased in the sh-RBP-J group compared to those in the MI group and the sh-NC group (*P* < 0.05, Figure 2B), which is consistent with the results of HYP content examined by ELISA (*P* < 0.05, Figure 2C). Additionally, we employed qRT-PCR to detect the expression patterns of fibrosis-related factors, which demonstrated that the expression levels of TGF-β1, PDGF-B, COL1, and COL3 in the heart were all significantly decreased in the sh-RBP-J group (all *P* < 0.01, Figure 2D). Together, these findings suggested that blockade of the Notch signaling pathway promoted the polarization of cardiac macrophages to M2, reduced the degree of cardiac fibrosis, and further validated that Notch signaling regulated macrophage polarization to influence fibrotic remodeling after MI.

### Blockade of Notch Signaling Reduced Cardiac Inflammation

**Figure 3:**
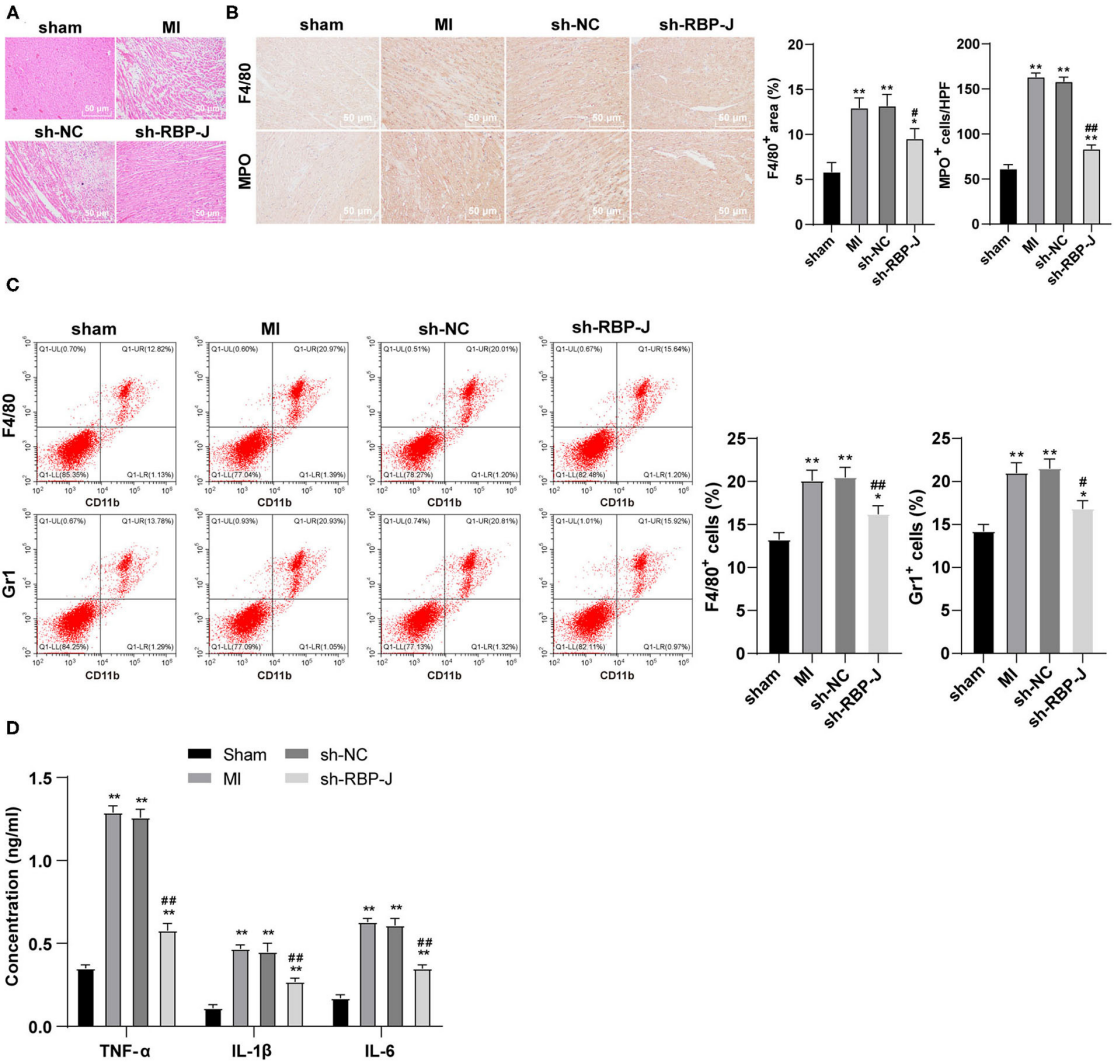
Blocking the Notch signaling reduced cardiac inflammation. **(A)**: HE staining was conducted to observe the mouse heart, *N* = 8; **(B)**: Immunohistochemistry staining was conducted to observe the macrophage and neutrophil infiltration in the mouse heart, *N* = 6; **(C)**: Flow cytometry was performed to observe the macrophage and neutrophil infiltration in the mouse heart, *N* = 6; **(D)**: ELISA was performed to detect the expression of inflammatory cytokines TNF-α, IL-1β, and IL-6 in the mouse heart, *N* = 6; three independently repeated tests were performed, and the data were expressed as mean ± SD. One-way ANOVA was used for analysis of variance, and Tukey's multiple comparisons test was used for *post-hoc* test; ** *P* < 0.01, * *P* < 0.05 (compared with sham group), and ## *P* < 0.01, # *P* < 0.05 (compared with sh-NC group).

Hematoxylin–eosin staining and immunohistochemistry staining were adopted to observe the inflammatory response in mouse hearts, such that the results of HE staining illustrated the presence of less inflammatory cells in the sh-RBP-J group than those in the MI group and sh-NC group, suggestive of a marked improvement in inflammatory levels and myocardial necrosis (Figure 3A). In addition, the results of immunohistochemical staining demonstrated that the infiltration of macrophages and neutrophils was significantly lower in the sh-RBP-J group than that in the MI group and sh-NC group (*P* < 0.05, Figure 3B). Moreover, flow cytometry results also confirmed that the proportions of F4/80 + CD11b + macrophages and Grl + CD11b + neutrophils were significantly lower in the sh-RBP-J group than those in the MI group and sh-NC group (*P* < 0.05, Figure 3C). Additionally, ELISA was carried out to detect the expression patterns of inflammatory factors TNF-α, IL-1β, and IL-6, and the results showed that the aforementioned inflammatory factors were all significantly decreased in the sh-RBP-J group compared with those in the MI group and sh-NC group (all *P* < 0.01, Figure 3D). Collectively, these findings suggested that blockade of the Notch signaling pathway reduced cardiac inflammation, and preliminarily confirmed that Notch signaling mediated macrophage polarization by affecting the inflammatory response and then regulating fibrosis.

### Blockade of Notch Signaling Inhibited the Activation of the NF-κB Pathway

**Figure 4:**
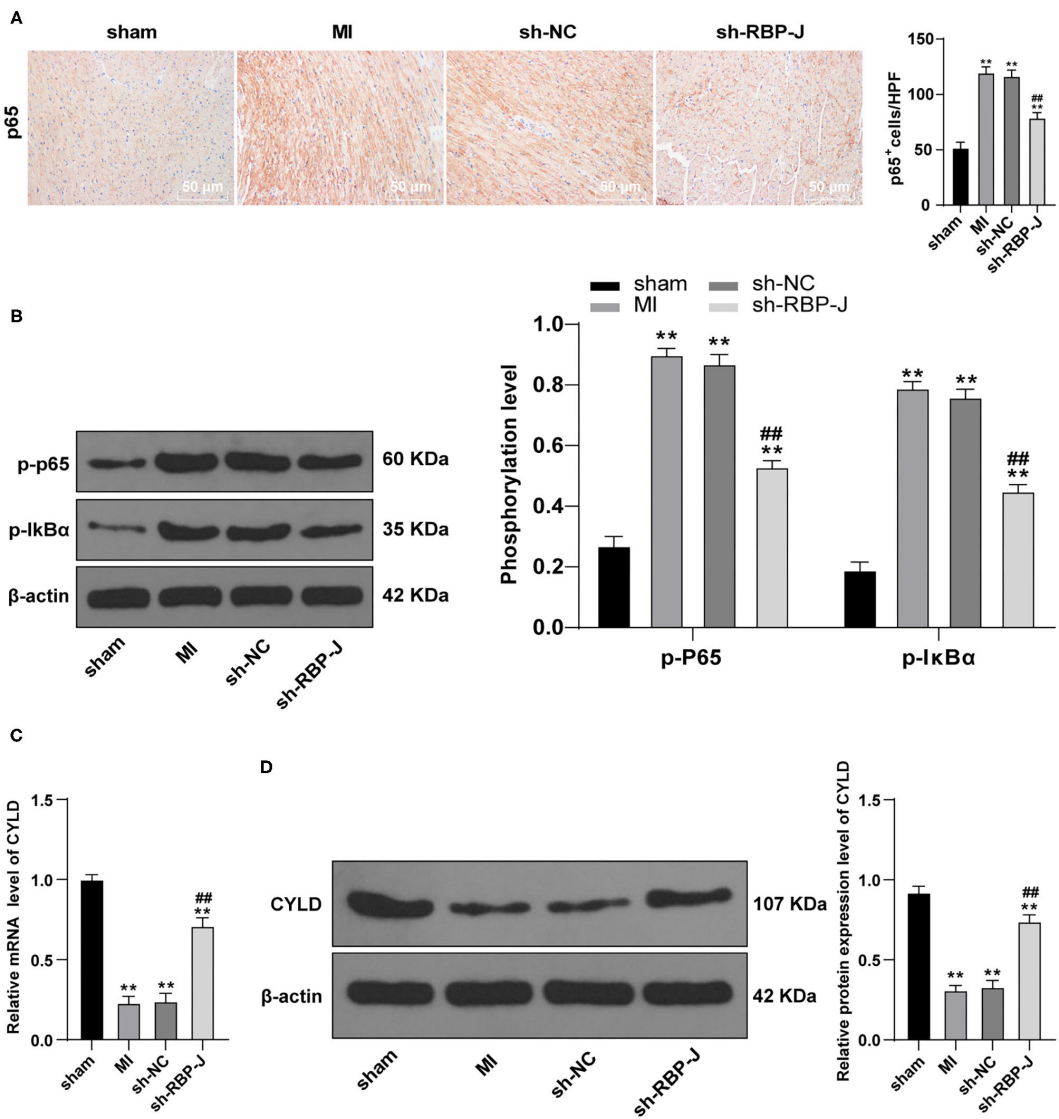
Blocking the Notch signaling inhibits the activation of the NF-κB pathway. **(A)**: p65 levels in mouse hearts were detected by immunohistochemistry staining; **(B)**: Phosphorylation level of the NF-κB pathway was detected by Western blot; **(C)**: mRNA expression of CYLD in mouse cardiac macrophages was detected by qRT-PCR; **(D)**: Protein expression of CYLD in mouse cardiac macrophages was detected by Western blot; *N* = 6; three independently repeated tests were performed, and the data were expressed as mean ± SD. One-way ANOVA was used for the analysis of variance, and Tukey's multiple comparisons test was used for *post hoc* test; ** *P* < 0.01 (compared with the sham group), and ## *P* < 0.01 (compared with the sh-NC group).

To further elucidate the specific mechanism of blocking the Notch signaling pathway to reduce cardiac inflammation levels, we carried out immunohistochemistry to detect p65 expression patterns in the myocardium. It was found that p65 levels were significantly decreased in the sh-RBP-J group compared with those in the MI group and sh-NC group (*P* < 0.01, Figure 4A). In addition, a Western blot assay was further performed to observe the changes in phosphorylation levels in the NF-κB pathway, the results of which illustrated that phosphorylation levels of p65 and IκBα were both significantly reduced in the sh-RBP-J group compared with those in the MI group and sh-NC group (all *P* < 0.01, Figure 4B), which suggested that blockade of the Notch signaling pathway inhibited the activation of the NF-κB pathway. Furthermore, recent studies have highlighted the involvement of CYLD in the regulation of the NF-κB pathway, which is further closely associated with cellular inflammation and fibrosis (16). Accordingly, we speculated that the mechanism by which Notch signaling mediated macrophage polarization to regulate fibrosis after MI might be related to CYLD. Subsequent detection of mRNA expression and protein levels of CYLD in cardiac macrophages by qRT-PCR and Western blot assay revealed that CYLD levels were significantly lower in the MI group and the sh-NC group than those in the sham group, while the sh-RBP-J group presented with partially restored levels of CYLD (all *P* < 0.01, Figures 4C,D). Altogether, these findings confirmed that the mechanism by which the Notch signaling mediated macrophage polarization to regulate fibrosis after MI was related to CYLD.

### Blockade of the Notch Signaling Pathway *in vitro* Promoted M2 Macrophage Polarization and CYLD Expression and Inhibited Fibrosis-Related Factors Expression and Cellular Inflammatory Levels

**Figure 5:**
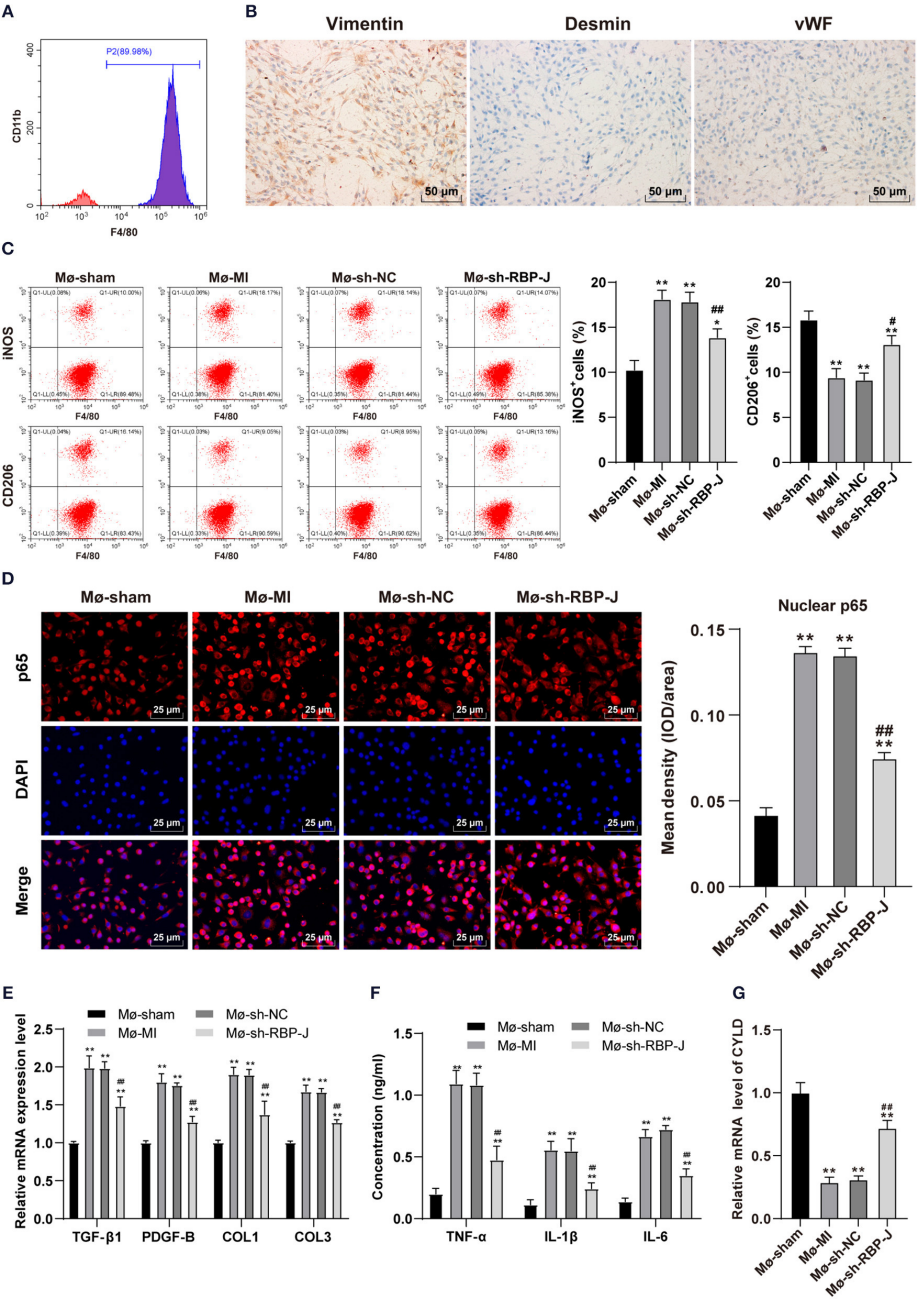
Blocking of the Notch signaling pathway *in vitro* promoted M2 macrophage polarization and CYLD expression and inhibited fibrosis-related factors expression and cellular inflammatory levels. **(A)**: Bone marrow-derived macrophages in mice were identified by flow cytometry; **(B)**: Heart-derived fibroblasts were identified by immunohistochemistry. Macrophages with different treatments were isolated and divided into Mø-Sham, Mø-MI, Mø-sh-NC, and Mø-sh-RBP-J groups. Macrophages and fibroblasts stimulated with LPS + INF-γ for 24 h were co-cultured by Transwell to establish an *in vitro* inflammatory model; **(C)**: Macrophage polarization was detected by flow cytometry; **(D)**: p65 in macrophage nuclei of each group was detected by immunofluorescence; **(E)**: Expression of fibrosis-related factors TGF-β1, PDGF-B, COL1, and COL3 in fibroblasts was detected by qRT-PCR; **(F)**: Expression of inflammatory factors TNF-α, IL-1β, and IL-6 in macrophages was detected by ELISA; **(G)**: The expression of CYLD in macrophages was detected by qRT-PCR. Three independently repeated tests were performed, and the data were expressed as mean ± SD. One-way ANOVA was used for analysis of variance, and Tukey's multiple comparisons test was used for *post-hoc* test; ** *P* < 0.01, * *P* < 0.05 (compared with Mø-Sham group), and ## *P* < 0.01, # *P* < 0.05 (compared with Mø-sh-NC group).

To further explore the mechanism by which Notch signaling mediated macrophage polarization and influenced fibroblast activation, we isolated and cultured primary bone marrow-derived macrophages and cardiac-derived fibroblasts and identified macrophages using flow cytometry. Subsequent results demonstrated that the isolated macrophages presented with highly expressed F4/80 +, which confirmed them as macrophages (Figure 5A). Afterward, the isolated fibroblasts were identified by immunohistochemistry, and the expression levels of vimentin were found to be positive (98%) in isolated fibroblasts, suggestive that the cells were derived from mesenchymal cells. In addition, we found that desmin expression was negative, basically excluding vascular smooth muscle cells; whereas, vWF antigen expression was negative, basically excluding endothelial cells (Figure 5B), and the results validated the collection of ideal cardiac fibroblasts. Macrophages and cardiac fibroblasts isolated and purified from the hearts of mice with MI in each group were then co-cultured with Transwell to establish an *in vitro* inflammatory model (LPS + INF-γ stimulated cardiac fibroblasts for 24 h). Macrophage polarization was subsequently detected by means of flow cytometry, the results of which illustrated that macrophages were polarized toward the M2 type (Figure 5C). Nuclear p65 in each group was also measured, and p65 levels in the sh-RBP-J group were found to be significantly lower than those in the MI group and sh-NC group (*P* < 0.01, Figure 5D). In addition, we carried out qRT-PCR to detect the expression patterns of TGF-β1, PDGF-B, COL1, and COL3 in cardiac fibroblasts, while ELISA was adopted to detect the expression patterns of inflammatory factors TNF-α, IL-1β, and IL-6 in macrophages. Subsequent results illustrated that the levels of fibrosis-related factors and inflammatory factors in macrophages were both significantly lower in the sh-RBP-J group than those in the MI group and sh-NC group (all *P* < 0.01, Figures 5E,F). Moreover, CYLD expression patterns in macrophages of each group were determined by qRT-PCR, the results of which demonstrated that mRNA expression levels of CYLD were significantly higher in the sh-RBP-J group than those in the MI group and sh-NC group (all *P* < 0.01, Figure 5G). Together, these findings confirmed that blockade of the Notch signaling pathway *in vitro* promoted macrophage polarization to the M2 type and the expression levels of CYLD, and inhibited the expression of fibrosis-related factors and cellular inflammation.

### Inhibition of CYLD Expression Reversed the Effects of Notch Signaling Pathway Blockade

**Figure 6:**
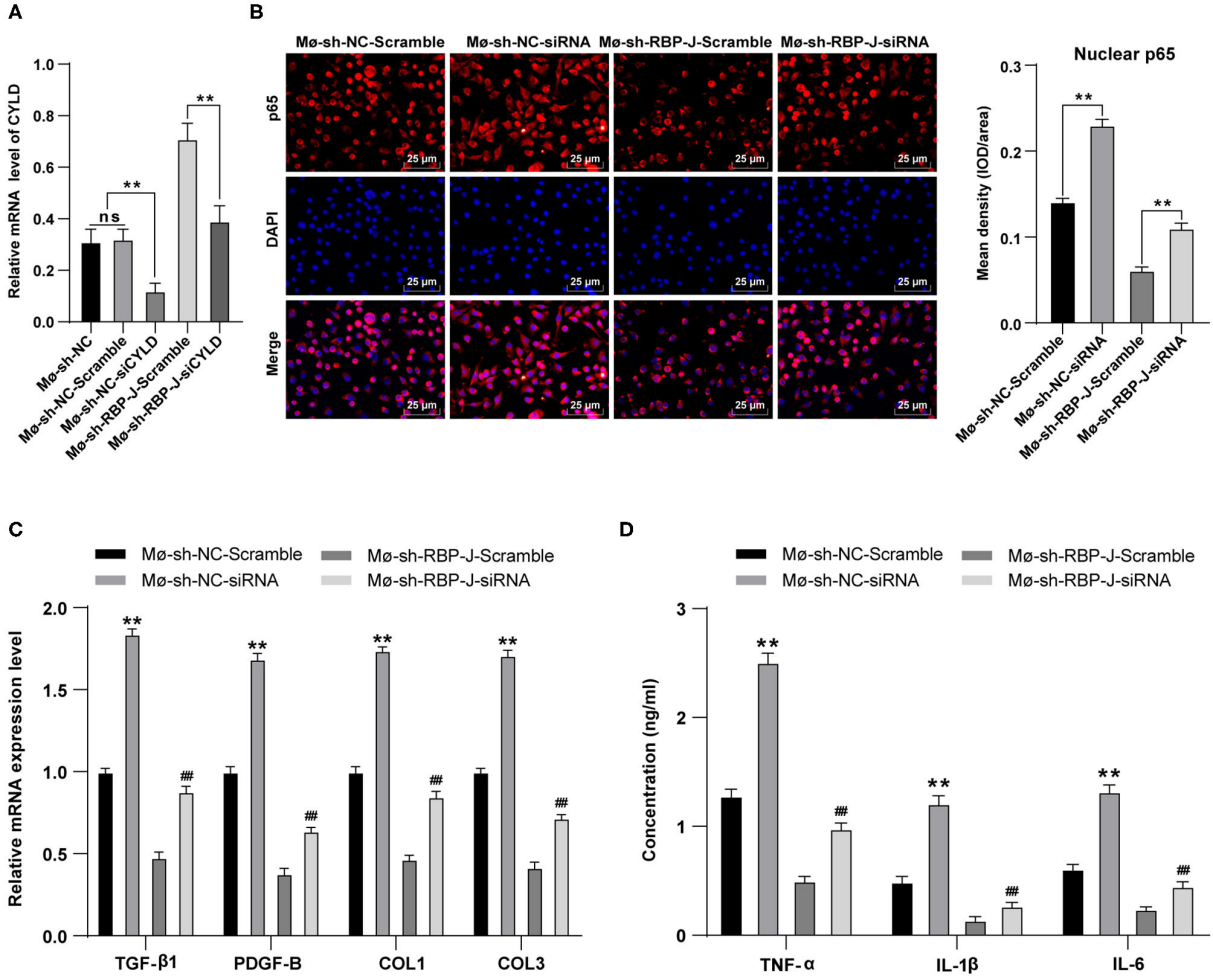
Inhibition of CYLD expression reversed the results that blocking of the Notch signaling pathway inhibited fibrogenesis and the release of inflammatory factors. siRNA and siRNA Scramble of CYLD were transfected into bone marrow-derived macrophages Mø-sh-NC group and Mø-sh-RBP-J group and stimulated with LPS + INF-γ for 24 h. **(A)**: mRNA expression of CYLD in macrophages of each group was detected by qRT-PCR; **(B)**: p65 in macrophage nuclei was detected by immunofluorescence; **(C)**: Expression of fibrosis-related factors TGF-β1, PDGF-B, COL1, and COL3 in fibroblasts was detected by qRT-PCR; **(D)**: Expressions of inflammatory factors TNF-α, IL-1β, and IL-6 in macrophages were detected by ELISA; Three independently repeated tests were performed, and the data were expressed as mean ± SD. One-way ANOVA was used for the analysis of variance, and Tukey's multiple comparisons test was used for *post- hoc* test; ** *P* < 0.01 (compared with Mø-sh-NC + Scramble group), and ## *P* < 0.01 (compared with Mø-sh-RBP-J + Scramble group).

**Figure 7:**
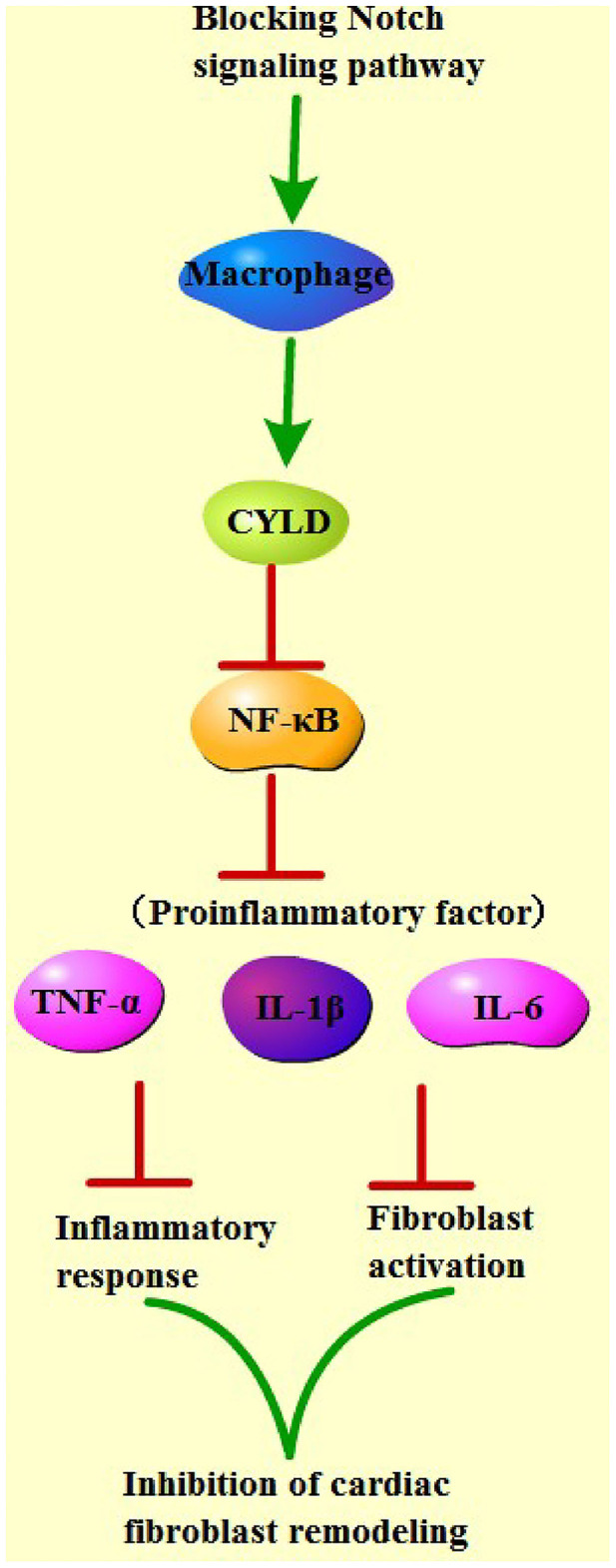
Mechanistic map of blocking the Notch pathway to improve cardiac fibrosis. Blocking of the Notch signaling pathway promoted M2 macrophage polarization and increased CYLD expression in macrophages, followed by inhibiting the activation of NF-κB pathway, inflammatory factors, and fibrosis-related factors, and ultimately suppressed cardiac fibrosis remodeling.

Finally, to determine the role of CYLD in the mechanism by which Notch signaling mediated macrophage polarization to regulate fibrosis after MI, we transfected siRNA and siRNA scramble of CYLD into bone marrow-derived macrophages of MI mice from each group. Subsequent detection of the mRNA expression patterns of CYLD in macrophages of each group by qRT-PCR revealed that CYLD expression levels were significantly lower in the sh-RBP-J-siCYLD group compared to those in the sh-RBP-J-Scramble group, whereas CYLD expression levels were significantly lower in the sh-NC-siRNA group than those in the sh-NC-Scramble group and sh-NC group (all *P* < 0.01, Figure 6A), suggestive of successful silencing of CYLD expression in macrophages. In addition, nuclear p65 levels were detected in each group, the results of which illustrated that the inhibition of CYLD expression significantly increased p65 levels in the nuclei of macrophages (*P* < 0.01, Figure 6B). Moreover, we determined the expression patterns of fibroblast-associated factors and inflammatory factors in the cells, which revealed that the expression levels of fibroblast-associated factors and inflammatory factors were both significantly increased after CYLD inhibition (all *P* < 0.01, Figures 6C,D). Altogether, these findings confirmed that blockade of the Notch signaling pathway promoted M2 macrophage polarization and increased the CYLD expression in macrophages, followed by inhibiting the activation of the NF-κB pathway, inflammatory factors, and fibrosis-related factors and ultimately resulting in the suppression of cardiac fibrosis remodeling (Figure 7).

## Discussion

Myocardial infarction is well known as a life-threatening cardiovascular disease associated with high mortality rates across the globe (2). Meanwhile, the process of macrophage polarization is known to play a critical role in cardiac remodeling after the occurrence of MI (6). Extensive investigations by our peers have further highlighted the involvement of the Notch pathway mediated through RBP-J in macrophage activation and plasticity (8, 9). Based on the aforementioned evidence, the current study carried out a series of experiments to explore the potential mechanism of the Notch signaling pathway in regulating macrophage polarization in fibrosis remodeling after MI.

First, we set out by establishing mouse models of MI, which were subjected to infection with adeno-associated virus-packaged RBP-J shRNA and adeno-associated virus-packaged sh-NC, followed by the assessment of mouse cardiac function. Subsequent findings demonstrated that RBP-J expression levels were significantly lower in the sh-RBP-J group compared to those in other groups, which suggested that hindering RBP-J in macrophages could block the Notch signaling pathway. Meanwhile, we also came across a significant reduction in the MI area of mice in the sh-RBP-J group relative to the MI and sh-NC group, indicating that blocking the Notch signaling pathway could improve myocardial injury in mice. Much in line with our findings, a previous study also documented the activation of the Notch pathway after infarction (13), which is indicative of the protective role conferred by the blockade of the Notch pathway in heart failure. Moreover, another study highlighted that the deletion of RBP-J in cardiomyocytes brought about increased hypoxia tolerance and improved cardiac function after MI (21), underscoring inhibition of RBP-J as a potential treatment strategy against ischemic injury. It is also noteworthy that recent investigations have insinuated that the depletion of Notch1 can ameliorate myocardial injury, while the miRNA, miR-146b, was previously illustrated to improve myocardial injury against inflammation *via* suppressing Notch1, which is in accordance with our results (22). Furthermore, we came across upregulated expressions of CD206 and downregulated expressions of CD11b in the sh-RBP-J group, which indicated that blockade of the Notch signaling pathway exerted a promotive effect on macrophage polarization to the M2 type. Additionally, further experimentation revealed that treatment with sh-RBP-J also brought about a decrease in collagen content and degree of fibrosis, which is particularly important as macrophages are heralded as highly significant in the process of fibrosis (23). Interestingly, there is a plethora of evidence to suggest the involvement of macrophages in MI, such that macrophage polarization is regarded as critical to cardiac remodeling after MI in humans (4, 6). Moreover, an insufficiency of macrophages was previously associated with impaired wound healing and promoted left ventricular remodeling after the occurrence of MI (24). Unsurprisingly, Notch signaling is also closely associated with macrophage polarization, such that blockade of Notch signaling can augment M2 polarization of macrophages (7). Collectively, we concluded that blockade of Notch signaling improved myocardial injury in mice, promoted macrophage polarization to the M2 type, and limited fibrosis.

The critical process of ventricular remodeling after MI is known to comprise several processes, mainly including inflammatory responses, fibrosis, and fibrosis remodeling. Thereafter, we further focused our efforts on verifying whether the Notch signaling could mediate macrophage polarization to modulate fibrosis by affecting the inflammatory response. Results of subsequent experimentation demonstrated that infiltration of macrophages and neutrophils in the sh-RBP-J group was significantly lower than that in the sh-NC group, in addition to a marked reduction in the inflammatory factors in the heart compared with those in the MI and sh-NC group. Corroborating our results, the study performed by Tsao *et al*. reported that knockdown of Notch1 could effectively reduce the inflammatory response in macrophages, a feat accomplished by decreasing TNF-α and IL-6 levels (25). There is also evidence to suggest that M2 macrophages can suppress extracellular matrix synthesis and inflammatory responses in the process of tissue repair (26). Meanwhile, Liu et al. recently demonstrated that Calhex231 can improve myocardial fibrosis after MI by regulating the autophagy-NLRP3 pathway in macrophages (4). Altogether, the aforementioned evidence supported our results such that blockade of Notch signaling exerts a reductive effect on cardiac inflammation and that Notch signaling-mediated macrophage polarization can regulate fibrosis by affecting the inflammatory response. It is also important to pay heed to the close association of the NF-κB signaling pathway with inflammation (27). For instance, Yoshiaki Sunami et al. previously uncovered that NF-κB signaling activation promoted liver fibrosis using macrophage-mediated inflammation (23). Furthermore, another prior study indicated that Notch was capable of regulating the NF-κB signaling pathway through the deubiquitinase CYLD in the immunoregulation of T cells (28). In addition, the activation of Notch signaling was previously shown to enhance NF-κB-mediated inflammatory response by downregulating CYLD expression and leading to aggravated hepatic ischemia-reperfusion injury (17). Similarly, it is no surprise that CYLD was previously implicated in the regulation of the NF-κB pathway and further associated with cellular inflammation and fibrosis (16), whereas CYLD is also known to negatively regulate the NF-κB pathway in high glucose-induced inflammatory models (29). Herein our study, we uncovered that blocking the Notch signaling pathway could inhibit the activation of the NF-κB pathway. Subsequent detection of CYLD expression patterns in macrophages using qRT-PCR illustrated that mRNA expression levels of CYLD were significantly higher in macrophages from the sh-RBP-J group compared to those in the sh-NC group. Moreover, we also learned that the inhibition of the CYLD expression brought about a significant promotive effect on the expressions of both fibroblast-associated factors and inflammatory factors. Collectively, these findings and evidence suggested that blockade of Notch signaling *in vitro* promoted macrophage M2 polarization and CYLD expressions and further inhibited the expression of fibrosis-related factors and cellular inflammatory levels.

In summary, findings uncovered in our study established that blockade of the Notch signaling pathway promoted M2 polarization of macrophages; increased CYLD expressions in macrophages; and inhibited the activation of the NF-κB pathway, inflammatory factors, and fibrosis-related factors, ultimately resulting in the suppression of cardiac fibrosis remodeling. However, we failed to investigate the underlying mechanism of macrophage polarization mediating myocardial remodeling and whether knockdown of RBP-J may promote healing by affecting the function of heart-related cells. Nevertheless, we shall explore these deficiencies in future endeavors in our sustained efforts to improve diagnostic and therapeutic approaches against cardiac remodeling in MI.

## Data Availability Statement

The raw data supporting the conclusions of this article will be made available by the authors, without undue reservation.

## Ethics Statement

The animal study was reviewed and approved by Animal Ethics Committee of General Hospital of Northern Theater Command.

## Author Contributions

ZL contributed to the study concepts, study design, and definition of intellectual content. ZL and MN contributed to the literature research. ZL, MN, and LY contributed to the manuscript preparation. HW contributed to the manuscript editing and review. MN, LY, DT, and QW contributed to the clinical studies. ZL, YH, YL, and HW contributed to the experimental studies and data acquisition. YZ and HH contributed to the data analysis and statistical analysis. All authors read and approved the final manuscript.

## Funding

This study was supported in part by a Grant-in-Aid from National Natural Science Foundation of China (Grant No. 81600311), Postdoctoral Science Foundation of China (Grant No. 2016M593030), Doctoral Scientific Research Foundation of Liaoning Province in China (Grant No. 201601402), and Liaoning Province, Science and Technology public relations project of China (Grant No. 201602805).

## Conflict of Interest

The authors declare that the research was conducted in the absence of any commercial or financial relationships that could be construed as a potential conflict of interest.

## Publisher's Note

All claims expressed in this article are solely those of the authors and do not necessarily represent those of their affiliated organizations, or those of the publisher, the editors and the reviewers. Any product that may be evaluated in this article, or claim that may be made by its manufacturer, is not guaranteed or endorsed by the publisher.
